# Inter-turn intervals in *Paramecium caudatum* display an exponential distribution

**DOI:** 10.1080/19420889.2024.2360961

**Published:** 2024-05-31

**Authors:** Sudhakar Deeti, Winnie Man, Johannes J. Le Roux, Ken Cheng

**Affiliations:** School of Natural Sciences, Macquarie University, Sydney, Australia

**Keywords:** Avoidance reaction, chemokinesis, chemotaxis, eukaryote, navigation, random-rate process

## Abstract

In navigating to a better location, mobile organisms in diverse taxa change directions of travel occasionally, including bacteria, archaea, single-celled eukaryotes, and small nematode worms such as *Caenorhabditis elegans*. In perhaps the most common form of goal-orientated movement, the rate of such turns is adjusted in all these taxa to ascend (or descend) a chemical gradient. Basically, the rate of turns is reduced when the movement results in better conditions. In the bacterium *Escherichia coli* and in *C. elegans*, the turns are generated by random-rate processes, in which the probability of a turn occurring is constant at every moment. This is evidenced by a distribution of inter-turn intervals that has an exponential distribution. For the first time, we examined the distribution of inter-turn intervals in the single-celled eukaryote, *Paramecium caudatum*, in a class exercise for first-year university students. We found clear evidence for an exponential distribution of inter-turn intervals, implying a random-rate process in generating turns in *Paramecium*. The exercise also shows that university laboratory classes can be used to generate scientific data to address research questions whose answers are as yet unknown.

## Introduction

Many mobile organisms show directed movements often called orientation or navigation [1–6], including microscopic organisms [3,4,6,7]. A common form of orientation – perhaps the most common – is *chemotaxis* [3,4,8,9], found in many forms of micro-organisms, including both prokaryotes and eukaryotes, as well as animals with nervous systems. The predominant form of chemotaxis is *chemokinesis*. In chemotaxis, an organism tracks the concentration of some chemical gradient in the environment and reacts to an increase or decrease in that chemical concentration. In chemotaxis proper, the choice of a new direction of travel likely hits upon a better direction for ascending or descending the gradient. In the more common chemokinesis, on the other hand, the choice of a new direction is random, while the rate of some behavior is altered as a function of changes in chemical concentration, such a change being a defining feature of kinesis. In *Paramecium*, our study organism, a turn takes place when an individual disengages the coordinated beating of cilia on its outside that generates forward movement. The organism turns a random amount in the medium before the cilia are reengaged in generating forward movement in a new direction [4,8,10]. How can random changes in direction result in directed movement? By adjusting the rate of directional changes appropriately [4,8–10]. Take gradient ascent as an example. When things get better, meaning that the gradient has a higher concentration of the tracked chemical than a moment ago, the rate of interruptions to forward movement accompanied by a random change of direction is decreased. When things do not get better, the rate of random changes in direction is increased. This basic chemokinetic servomechanism has been identified in bacteria (*Escherichia coli* [9]; *Salmonella enterica*: [11]), Archaea [12], eukaryotic *Paramecium* [8], and nematode worms (*Caenorhabditis elegans*: [13,14]). Chemokinesis drives all these taxa to the peak of a chemical gradient [4]. There, things do not get better (the gradient does not increase), the organism turns often, and ends up milling around the area of optimal conditions. Chemokinesis is used to ascend food gradients and descend aversive gradients.

Our account concerns the timing of such random changes in direction in *Paramecium*, for which we understand the form in *E. coli* and *C. elegans*, but not in Archaea or single-celled eukaryote *Paramecium*. By understanding, we mean finding the function that best fits the distribution of inter-turn intervals, turns variously called tumbles in *E. coli* [9], avoidance reactions in *Paramecium* [8], and pirouettes in *C. elegans* [13]. In *E. coli* [15] and in *C. elegans* [14], the distribution is best fit by an exponential function (with a negative exponent). The distribution is highly asymmetric, bunched at the left, short end, and dropping quickly at the right tail. Exponential inter-event intervals, of which inter-turn intervals are an example, form a signature for a random-rate process [4], i.e. when at every moment in time there is an equal and constant probability of the event (such as a turn) taking place, giving us a clue to the generative mechanism underlying the process. No other explanation is plausible given this signature. If non-neural bacteria use a random-rate process and the neurally endowed (albeit minimally) *C. elegans* also does so, then what about eukaryotes such as *Paramecium*?

While piles of data on the times at which *Paramecium* made turns sit on laboratory shelves, we took a many-hands approach to gather the data in a practical session in a first-year biology class at Macquarie University, *Genes to Organisms*. Hundreds of students watched the movement of *Paramecium* specimens on a microscope slide for 2–3 minutes and recorded the times at which their organism turned. On most occasions, the specimens turned in the medium without bumping into any obstructions such as debris; the inter-turn intervals thus reflect outputs of endogenous processes generating turns. While the use of observers lacking experience no doubt reduced the precision of the data, the large sample size made up for this deficiency. As the class was not told the hypothesis of what to expect, we do not think that systematic errors would be introduced, only non-systematic errors that would not bias the data to favor one function over another.

We fitted three functions to the obtained distribution of inter-turn intervals. Following work on the inter-event intervals in scanning in desert ants [16] (more on this in the Discussion), these were the exponential function, already described, the power law function, and a stretched exponential function thought to be a generalized form of an exponential function. The fits of these functions were evaluated using Akaike Information Criterion.

## Methods

### Experimental background and study species

Data were collected as part of an undergraduate unit, BIOL1110 *Genes to Organisms*, at Macquarie University, Sydney, Australia, in 2023. In a practical class offered in this unit, students (listed in supplementary materials) learned about cellular functioning and physiology, by observing the workings of organelles, such as the nuclei, contractile vacuoles, cytopharynges, and cilia in *Paramecium caudatum*. Students were given a background lecture on the basic biology of *Paramecium caudatum,* such as its feeding, movement, and reproduction, prior to data collection.

*Paramecium caudatum* cultures were obtained from Southern Biological (Alphington, Victoria, Australia). The culture came in 100-ml jars, and five jars were purchased. When the jars arrived, the lids were removed, and the *Paramecium* was kept in the dark in an air-conditioned teaching laboratory at Macquarie University at ~ 20°C. *Paramecium* cultures can be kept for a month in these conditions.

### Experimental procedure

Students worked in pairs and each pair prepared a microscope slide by placing one drop of *Paramecium*-containing suspension and one drop of the methyl-green/pyronine dye on a slide. The *Paramecium* culture and dye were mixed gently by placing a coverslip on the slide. Students observed the prepared slides under 10X magnification using a Motic BA210 compound microscope with a Moticam 2.0MP camera, running with the software Motic Images Plus 3.0 (×64) and were tasked to focus on a single *P. caudatum* individual. Demonstrators made sure that all students were tracking a *P. caudatum* individual and not any other organisms such as *Euplotes* (protozoa) or microannelids. Once a *P. caudatum* individual was identified and in focus, students used the Motic Images Plus 3.0 software to record a 2–3 minute-long video, while moving the microscope table to keep the object in the field of view. An example of a video and information on how to score the intervals between *P. caudatum* turning events were provided to all students prior to data collection. Briefly, each pair of students watched the video recording and at the first turning event (of 90 degrees or more) of the *P. caudatum* individual, one student kept time using the “lap” function on a smartphone, while the other student observed the movement of the organism. At the second turning event the observer alerted the timekeeper, and a “lap” time was recorded again. Laps were recorded for every turning event until the end of the video recording (*n* = 250 video recordings). In this fashion, all the times of turns were recorded on a spreadsheet.

### Data analysis

From 250 samples of *Paramecium* records, the inter-turn intervals were binned for display. Fitting binned data, however, introduces errors [17], so that curve-fitting was based on cumulative frequency distributions, with inter-turn intervals (duration) on the *x*-axis and cumulative turn numbers (frequency) on the *y*-axis. Given the highly left-skewed data, we fitted three plausible functions, following Deeti et al. [16]: power-law, exponential, and stretched-exponential functions.

The power law (y = α * t^β^) had free parameters α, β. α is a scaling constant, β is an estimate of the power exponent in the power law, and t is the inter-turn interval (x values). We logged both x and y observation values and proceeded to build a power law prediction model. In this model, we calculated the cumulative sum of the y vector values and applied the inverse on the x observation values.

The exponential model (y = α * e^βt^) had free parameters α, β. α is a constant which scales the function and β is a free parameter estimating the negative exponent, while t represents turn interval. We logged only the y observation values. We defined and applied an exponential equation function with defined parameters to build the exponential model.

The stretched exponential is fβ (t) = α * e^(–t^β^), where ^ means “to the power of”. The approach applied in fitting a stretched exponential is similar to that used for fitting an exponential equation. The difference is that to solve stretched exponential, we needed to pre-define a set of functions for our parameters. We set a starting value for α, β, and t which can then be adjusted to fit our data. Whereas β is the stretching parameter (0 ≤ β < 1), fβ(t) represents the decay of observables with change in predictive parameter (differential distribution), with t representing duration, the predicting variable. For the stretched exponential, we used nonlinear modeling. We logged only the y observation values and defined an equation to obtain the appropriate α, β, and t parameters of the data. We fitted the data using the Levenberg-Marquardt algorithm to obtain the values that produced the maximum likelihood. We built a preliminary model using the Nonlinear Minimization (NLM) function and a second model using the Nonlinear Least Squares (NLS) function, which used the obtained parameters as a starting point to achieve the best log-likelihood of the fitted model data.

The best model distribution was objectively identified using R-squared (*R*^2^), Akaike information criteria (AIC), p-Value, and AIC weight. A linear model was used to verify that the fit to the models could not be rejected at *p* < .05. These metrics provided insights into the goodness of fit and helped determine the most appropriate model for our dataset.

## Results

**Figure 1.**
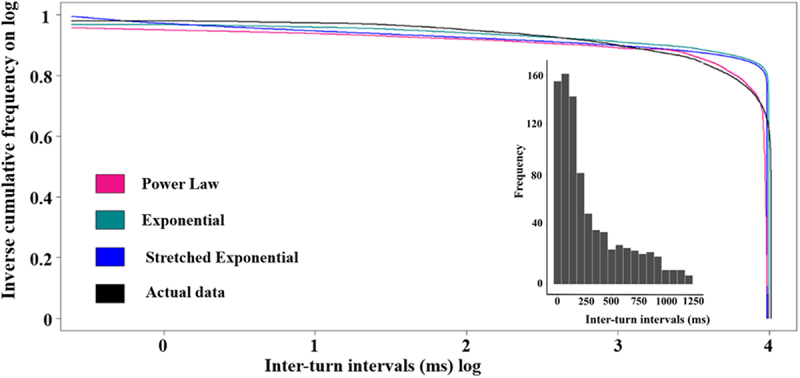
Distributions of inter-turn intervals. The smoothed inverse frequency distributions (in black) of inter-turn intervals, with 3 models fitting the distribution: Power law (pink), Exponential (green), Stretched Exponential (blue). The observed data have been smoothed using symmetric Gaussian smoothing, but the curve-fitting was based on the raw, unsmoothed data. The x- and y-axis measures are on a log scale. The inset figures shows the binned frequency distributions of inter-turn intervals.

The inter-turn interval data, combined from all samples of movies of *Paramecium caudatum* moving on slides (for a representative clip, see Supplemental materials: https://osf.io/34dtv/files/osfstorage/664c4b6e7250fa1b4f4e796c), exhibited a left-skewed distribution. The binned frequencies for these intervals declined quickly, the decline being more gradual beyond 250 inter-turn intervals (Figure 1 inset).

We fitted the cumulative data using the power law, exponential, and stretched exponential functions (Figure 1, Table 1). To eyeball the fits, one should focus on the bulk of the data in the flat part of the distribution and not focus on the right tail where the data are sparse. Both the power law and the exponential models fitted well, with high *R*^2^ values (0.999077 and 0.99942, respectively) and being highly significant (*p* < .0002). The exponential model, however, demonstrated the best overall performance, achieving by a margin the lowest AIC value and an AIC weight of 1, indicating its superior explanatory power among the models considered. The stretched exponential model, even though providing a significant fit (Table 1) exhibited a lower *R*^2^ value (0.978865) and a much higher AIC value of 24,143.4, suggesting a less optimal fit to the data. In sum, these findings show that the exponential function provides the most parsimonious and accurate representation of the underlying data.

**Table 1.** The performance of curve fits of inter-turn intervals. AIC = Akaike Information Criterion. Curve fits were performed using the maximum-likelihood method on the inverse cumulative distribution.

Data Model	AIC	R Squared	P Value	AIC Weight
Power Law	16748.03	0.999077	<2.2e-16	0.00
Exponential	13997.04	0.99942	<2.2e-16	1
Stretched Exponential	24143.4	0.978865	5.361e-06	0.00

## Discussion

Our results from a practical exercise in a first-year biology class showed that the distribution of inter-turn intervals in *Paramecium caudatum* is fitted best by an exponential function. We base this conclusion on the AIC weights, which overwhelmingly favor the exponential model. Even though the *R*^2^ values of the power law model and the exponential model are very similar, the variance unaccounted for (1 – *R*^2^) is 59% higher for the power law model. The AIC is evidently sensitive to this difference. The results of the exercise have biological and educational significance, and we comment briefly on each.

An exponential distribution of inter-event intervals has only one reasonable interpretation: the events are generated by a random-rate or Poisson process [4,14,15]. In a random-rate process, the chance of the event taking place is constant at every moment in time, in a sense making the timing of the events as random as possible. The word “constant”, however, only applies to short moments in time. The constant can also be considered as a free parameter in the process, one to some extent under the control of the organism. *Paramecium* adjusts the rate of turning according to environmental feedback in chemokinesis [4,8]. We can now say that in chemokinesis, *Paramecium* adjusts the rate parameter that governs the random-rate process generating turns, much like a range of other taxa. These other taxa that display random-rate processes in changes of direction include bacteria (*E. coli*: [15]), nematode worms with only 302 neurons (*C. elegans*: [14]), and desert ants in various aspects of the stop-and-look behavior that they exhibit occasionally in navigation [16]. Bouts of scanning, durations of those bouts, and the duration of individual fixations all show exponential distributions in their inter-event intervals [16]. The use of random-rate processes seems widespread across mobile life. The full significance of these comparative findings remains to be fully explored and explained.

Results from our exercise also show that university classes can produce experimental results that are worth publishing even though the students were not experts or even experienced with the study organism. Some of our student participants expressed excitement over this aspect of their practical exercise and being able to contribute to “real science”. The results might lack some reliability or precision, but in the current case, it is inconceivable that highly unreliable or imprecise data could produce a pattern of data that so cleanly supports a formal, mathematically formulated hypothesis. Perhaps future classes could assess both the reliability and the precision of the *Paramecium* data gathered for the current study, as well as test the replicability of our results.

The BIOL1110 practical exercise is, of course, not the first time that a class has been used to generate scientific data. This is not the place for a review of this topic, but we end with two examples. The last author has used another first-year biology class (*Biological Basis of Behaviour*) to test the effect of drawing on learning concepts in the class, a ‘field experiment’ in educational psychology [18]. Classes at university or college level are not necessary. One study featured an experiment conducted by primary school children on learning in honeybees [19], in a unique paper with hand-drawn graphs and no references. Such real science can not only produce scientific results but also inspire students to become scientists. We can do no better than to end by quoting Blackawton et al. [19], who wrote:

Real science has the potential to … transform the way one thinks of the world and oneself. (p.168)

## Supplementary Material

Supplemental Material

## Data archival

The raw data and analysis code from this study are archived at: Open Science Framework https://osf.io/34dtv/files/osfstorage.
