# Supplementary material for: Inter-turn intervals in Paramecium caudatum display an exponential distribution
Source: Commun Integr Biol. 2024 May 31;17(1):2360961. doi: 10.1080/19420889.2024.2360961 (PMC11146437; doi:10.1080/19420889.2024.2360961)
Supplement: Supplemental Material [file KCIB_A_2360961_SM9279.zip › Supplementary methods.docx]

**Supplementary methods**

**Inter-turn intervals in *Paramecium caudatum* display an exponential distribution**

Sudhakar Deeti, BIOL1110 Class2023, Winnie Man, Johannes J. Le Roux, Ken Cheng^3^

Three functions were fitted to the inverse cumulative data of inter-turn intervals, the power law, the exponential function, and the stretched exponential function.

The power law (y = α * t^β^) had free parameters α, β. α is a scaling constant, β is an estimate of the power exponent in the power law, and t is the inter-turn interval (x values). We logged both x and y observation values and proceeded to build a power law prediction model. In the power law model, we calculated the cumulative sum of the y vector values and applied the inverse on the x observation values.

The exponential model (y = α * e^βt^) had free parameters α, β. α is a constant which scales the function and β is a free parameter estimating the negative exponent, while t represents turn interval. We logged only the y observation values. We defined and applied an exponential equation function with defined parameters to build the exponential model.

The stretched exponential is fβ (t) = α * e^(–t^β^), where ^ means “to the power of”. The approach applied in fitting a stretched exponential is similar to that used for fitting an exponential equation. The difference is that to solve stretched exponential, we needed to pre-define a set of functions for our parameters. We set a starting value for α, β and t, which can then be adjusted to fit our data. Whereas β is the stretching parameter (0 ≤ β < 1), fβ(t) represents the decay of observables with change in predictive parameter (differential distribution), with t representing duration, the predicting variable. For the stretched exponential, we used nonlinear modelling. We logged only the y observation values and defined an equation to obtain the appropriate α, β, and t parameters of the data. We fitted the data using the Levenberg-Marquardt algorithm to obtain the values that produced the maximum likelihood. We built a preliminary model using the Nonlinear Minimization (NLM) function and a second model using the Nonlinear Least Squares (NLS) function, which used the obtained parameters as a starting point to achieve the best log-likelihood of the fitted model data.
